# Mycotoxin source and its exposure causing mycotoxicoses

**DOI:** 10.6026/97320630019348

**Published:** 2023-04-30

**Authors:** Alshammari Nawaf

**Affiliations:** 1 Department of Biology, College of Sciences, University of Hail, Hail, Saudi Arabia

**Keywords:** Mycotoxin's, fungus, environment, toxins

## Abstract

Mycotoxins are toxic compounds produced by fungi such as *Aspergillus*, *Penicillium*, *Rhizopus*, *Fusarium* spp., and mushrooms. They are present in the mycelium or in the spores of the fungus. They cause human health problems once ingested. This is common in countries with high ambient temperature and relative humidity such as in the tropical regions. The consumption of moldy food and feeds are injurious to people and animals. The linked acute and chronic diseases target organs in humans and animals. The clinical symptoms depend on the intrinsic toxic features of the mycotoxin, the quantity, and length of exposure. The diseases caused by ingesting mycotoxins are reffred as mycotoxicoses. Therefore, it is of interest to document known data on the mycotoxin source and its exposure causing human hazards leading to mycotoxicoses.

## Background:

Fungi are defined as heterotrophic eukaryotic organism with a chitin us cell wall. They might be simple, single cell organisms or complex, multicellular ones. They thrive on animals, plants, and humans as they provide a ready source of nutrients. Fungi include the far more widespread yeast and molds. Mold and yeasts are types of fungi. Fungi are found in diverse habitats such as oceans, rocks, deserts, and salty environments. Humans can reap many benefits from fungi. For instance, we consume mushrooms for their high fiber and protein content and we also use different types of fungi in the production of staples bread, beer, and cheese. There are fungi that can cause harm to humans. Common example of harmful fungi include greenish-blue mold commonly found on bread [1]. If you encounter mold on food, it is best to throw it away because it may have been tainted. Despite their preference for warm and humid conditions for growth, fungi can grow in almost any temperature and on virtually any food. The types of fungi hazardous for consumption include molds, yeast, and mushroom. Molds are microscopic fungi that live on plant or animal matter. These spores can be transported by air, water, or insects. On the other hand, yeast is single celled fungi in contrast to molds that are multicellular. They differ from bacteria by their large cell size and shape, which may oval, elongated, elliptical, or spherical. The average cell size of yeast is from five to eight micrometers in diameter.

## Environmental factors in producing toxins:

There are certain conditions which are necessary for the fungi to produce hazardous amounts of toxins including:

## Moisture:

Pathogenic fungi require moisture content combined with a relative humidity of 85 percent to grow. In this case, the higher the moisture content the faster the fungus grows.

## Temperature:

 The minimum temperature for the production of aflatoxin is 12°C, the optimum is 27°C, and the maximum is 42°C. In this case, the fungus grows slowly below the minimum temperature, and will grow rapidly above the maximum temperature at which aflatoxin is produced. Furthermore, stored rice, corn, or wheat may experience a rise in temperature when *Aspergillus flavus* grows as a predominant organism.

## Presence of other micro flora:

Several funguses such as, *Aspergillus flavus* rarely invade the grains alone and requires the presence of other fungi. In this case, the grains must be moist enough to allow fungi invasion such as *Aspergillus candidus*, and the yeast like *Aspergillus flavus*. The metabolic activity of these fungi results in moisture content is raised to over 18 percent and temperature to 35°C or 40°C allowing for *Aspergillus flavus* taking over.

## Substrate:

In this case, given an equal growth of *Aspergillus flavus*, much more aflatoxin will be formed in one substrate than in another. For instance, even though *Aspergillus flavus* grows in both soybeans and peanuts, there is little aflatoxin produced in soybeans while peanuts record large contents of aflatoxin. Therefore, some food or feed materials have low or moderate aflatoxin risk, while others have high aflatoxin risk.

## Time:

Fungi produce a wide array of toxins to counteract predators and in other instance to minimize competition from other organisms [2]. This happens over a period of time.

## Mycotoxins:

Mycotoxins refer to the toxic compounds produced by fungi such as *aspergilli*, *penicillin*, *rhizopus*, *fusarium* spp., and mushrooms. They are produced by various strains of fungi including Aspergillus, Penicillum, Rhizopus, Fusarium spp., and mushrooms. Theycause health problems in human once ingested and are particularly common in countries with high ambient temperature and relative humidity such as in the tropical regions. Mycotoxins are present in the mycelium or in the spores of the fungus itself. Mycotoxins are hazardous to animals and human. The well-known examples of mycotoxin include Mycetism - Amatoxins and Phallotoxins, Aflatoxins, Coprine, Fumonisins, Deoxynivalenol (DON), Ochratoxins, Trichothecenes, Zearalenone and Gyromitrin.

## Mycetism - Amatoxins and phallotoxins:

Mycetism is also referred to as mushroom poisoning occurs when a person misidentifies and consumes poisonous mushrooms. In this case, most mushroom species produce gastrointestinal distress once consumed. *Amanita phalloides* is a poisonous mushroom species in which the fruiting body contains lethal doses of amanitins toxin. The mushroom is responsible for vast majority of known mushroom-poisoning deaths [3]. The species is mycorrhizal meaning that it lives in partnership with tree roots. The toxins from the mushroom target the liver and kidneys with the victim vomiting and feeling nausea, which is followed by seizures and coma that may lead to death. Amatoxins and phallotoxins are two mycotoxins form the two major groups of peptidic mushroom toxins. Toxic compounds belongs to the subgroup of nine related compound known as Amatoxins are restricted to three different types of poisonous mushrooms:*Amanita*, *Galerina*, and *Lepiota*. Amatoxins are lethal even in small doses often leading to death. The structure of the amatoxin consists of eight amino-acid residues arranged in a conserved macro-bicyclic motif [4]. Amatoxins inhibit RNA polymerase II, an enzyme used in the synthesis of messenger RNA (mRNA). In absence of mRNA, cells were not able to synthesize protein, as a result cell metabolism stops and lysis occurs. Amatoxins moves within the bloodstream to reach organs such as the liver and heart leading to fatalities. Phallotoxins get attached to the polymeric actin in order to stabilize microfilaments and also reduces monomeric actin in steadiness with the filaments [5]. Cells treated with membrane-permeable phalloidin derivatives showed signs of apoptosis, including shrinkage and the formation of blebs, when viewed under the microscope.

##  Aflatoxins:

Fungi of the genus *Aspergillus* that commonly on agriculturally important crops are known to produce toxins called as Aflatoxins.. Aflatoxin is named from *Aspergillus flavus*, where the 'a' comes from Aspergillus and the 'fla' comes from flavus. About 60 percent strains of *Aspergillus flavus* produce aflatoxin [6]. Aflatoxins are produced mainly by fungi *Aspergillus flavus* and Aspergillus parasitica. At room temperature, aflatoxins crystals range from nearly colourless to a very light yellow. They dissolve marginally in water and hydrocarbons, but they dissolve completely in polar solvent including methanol, acetone, and chloroform. There are four primary types of aflatoxins namely B-1, B-2, G-1, and G-2 named from their blue and green fluorescence, respectively, on thin-layer chromatography plates. Dairy feeds containing contaminated corn and cottonseed meal are the source of Aflatoxin M1 contamination in dairy products [7].

Humans and animals are exposed to aflatoxins primarily by consuming contaminated food. Inhalation of dust created by working with infected crops and feeds is the main route of occupational exposure to aflatoxins. When animals consume this contaminated feed having aflatoxin, it ultimately results in the contamination of milk, eggs, and meat. Aflatoxicosis is a disease caused by the consumption of aflatoxin. Aflatoxin B1 is considered as responsible for both toxicity as well as carcinogenicity. Human and animals are sensitive to poisoning by aflatoxin as extremely small amounts of the toxin consumed in food cause damage to internal organs such as the liver. Liver is the main target organ and liver damage occurs in poultry, fish, rodents, and non-human primates when they feed on foods contaminated with aflatoxin B1. Aflatoxin causes genetic damage in bacteria, in cultured cells from humans and experimental animals, and in human and experimental animals exposed to aflatoxin [8].

Various methods like thin-layer chromatography (TLC), high performance liquid chromatography (HPLC), gas chromatography along with fluorescence spectrophotometry and frontier infrared spectroscopy are used to to detect and quantify aflatoxins in agricultural crops and feeds [9]. Immunochemical methods rely on the specificity of binding between antibodies and antigens and include radioimmunoassay, enzyme linked immunosorbent assay, lateral flow devices, and immunosensors. Simple and inexpensive field screening methods help determine that the food and feed is free from aflatoxins. There are different methods employed for decontamination and detoxification of aflatoxin-laden foods. Physical methods include the separation of the contaminated fractions, removal, or inactivation by physical means such as heating, cooking, roasting, and radiation [10]. Dust, husks, and other materials that are explored by molds are all eliminated through sorting and rinsing during the cleaning process. Adsorption is used to lower levels of aflatoxin . Adsorption occurs in the digestive track bybinding the toxin compound to the adsorbent compound.. Absorbable compounds include active carbon, diatomaceous earth, alumino, complex carbohydrates located at plasma membrane of yeasts and bacteria (glucomannans, peptidoglycans), and synthetic polymers. After testing the efficacy of yeast, activated charcoal, and zeolite as aflatoxin absorbents in broiler diets, it was found that the combination was effective in decreasing the hallmarks of aflatoxin toxicity in developing broilers [11]. Chemical methods of decontamination and detoxification of aflatoxin-laden foods rely on chemical agents. Chlorinating agents such as sodium hypochlorite, chlorine dioxide, and gaseous chlorine degrade aflatoxins. Others include; oxidizing agent such as hydrogen peroxide, ozone, and sodium bisulphate. For hydrolytic agents and alkalis, they degrade aflatoxins by either oxidizing the double bond of the terminal furan ring or hydrolyze and oxidize the lactone ring of *Aspergillus flavus* B1. Biological methods of aflatoxin decontamination rely on bacteria, yeast, and fungi in solutions. The mycelia of aflatoxin producing molds can break down the toxin they produce with the help of peroxidase enzyme. Aflatoxins reacts with free radicals produced by peroxidase catalysing the decomposition of hydroperoxides [12].

Around one hundred thousand turkeys died after eating tainted ground nuts in 1960, marking the first high value mortality from. During the feeding process, they were fed with seeds that had been infected with fungus *Aspergillus flavus* and *Aspergillus parasiticus* [14]. In the presence of UV light, aflatoxin AFB1 and AFB2 look blue in color, whereas aflatoxin AFG1 and AFG2 appear green in color. Aflatoxins B1 and B2 is synthesize by *A. flavus* and *A. parasiticus*, however, on the other hand, aflatoxins G1 and G2 is synthesize exclusively by only *A. parasiticus*. When aflatoxins B1 and G1 are injected into animal feed, it results into the formation of M1 and M2 aflatoxins in milk. There is always an association between aflatoxin B1 and B2. Rice and nuts are the most common cereals infected by molds that produce aflatoxins. Molds can be inhaled or ingested into the body through oral routes through either inhalation or ingestion. The duodenum makes up a major portion of the intestinal tract and it is one of the major sites of absorption of these substances after they are swallowed in the gastrointestinal tract. By passive diffusion, it is able to reach into the blood without any extra effort on the part of the user [15, 16, 17].

Aflatoxin is one of the most studied mycotoxins. Aflatoxin B1 is a mycotoxin that has been reported to cause hepatocellular carcinoma in humans and animals as a class-1 carcinogen. Aflatoxins have compounds that are highly immunosuppressive, acutely toxic, , teratogenic, mutagenic, and had an ability to create tumors within their targets. There is a high risk of liver cirrhosis as a result of this condition. As a consequence of the ingestion of aflatoxin, rats with a high protein diet have a increased risk of liver cancer. Additionally, many other evidence hinted its involvement in causing breast cancer and can lead to the development of breast tumors [17]. There is a link between breast cancer and moldy cheese containing aflatoxin. The results of a very simple experiment showed that feeding animals with moldy rice alcoholic extract resulted in the development of breast cancer in the animals. The results of this simple experiment indicate that there is a relationship between mycotoxins and breast cancer in humans, although no specifics were provided [18]. In addition, AFB1 is capable of oxidizing into a number of toxic, carcinogenic and mutagenic compounds. DNA strands can be broken and point mutations can occur as a result of it. Aflatoxins are primarily eliminated from the body by urination, bile and in the case of nursing mother’s milk [19].

## Stigmatocystin (ST):

In order to protect our bodies against cancer, the p53 gene had a crucial role in defending us from the disease. Nonetheless, p53 gene is no longer able to control cancer when it gets mutated. In the past few years, research has shown that mycotoxins such as aflatoxin have been associated with DNA damage and have the potential to inactivate the p53 gene as well. Mutations in the p53 gene are caused by aflatoxin B1. It prevents the breakdown of glucose and glycogen. As a result of abnormal changes in this specific gene, the cell can proliferate out of control, resulting in cancerous growths [20, 21]. In *Aspergillus versicolor* and *Aspergillus nidulans*, sterigmatocystin is produced. Cheese, green coffee beans, and moldy stored grains have been reported to be sources of the disease. Bread cured in salt, ham and salami have been found to contain relatively high levels of sterigmatocystin. There is a high risk of infection through the consumption of moldy cheese crusts. In damp places, it is also common to notice moldy odors in the air which can cause irritations to the eyes, nose, throat and lungs, and it has been reported that its presence can cause moldy odours in the air as well. It is very common to find this type of problem on textiles, wall boards, insulation, and ceiling tiles that have been damaged by water. There is an occasional occurrence of this condition in carpet dust from an indoor environment [22, 23]. In contrast to aflatoxin, ST is much less commonly found. The mycotoxin is mutagenic, carcinogenic, and teratogenic. Its presence in foods is cause of concern because it can leads to hepatocellular cancer.. It is a toxin that is responsible for causing acute hepatitis, hemorrhages, Rey's syndrome, and proliferations of the bile ducts, although it is mostly removed during rice milling [24]. Besides cancer of the lungs and kidneys, it also causes a number of other diseases. Moreover, *A. versicolor* is an opportunistic pathogen, like many other members of its genus, and it is considered to be one of the most important causative agents of Aspergillosis in the world. It has a structural similarity to aflatoxins in that it causes tumors, however it is less lethal as compared to aflatoxins [25]. In human peripheral lymphocytes, ST levels as low as 10 mg/day, either fed continuously or given under the skin, cause DNA damage and apoptosis, when given at levels as low as 15 mg/day [26].

## Carcinogenicity of Aflatoxin B1(AFB1):

Ingestion of AFB1 leads to its absorption and transport from the small intestine to the liver [26]. Nonionic diffusion allows it to pass through hepatocytes without being affected by metabolic energy [24]. In the liver, cytochrome P450 (CYP450) metabolizes AFB1 into an epoxide called AFB1-E even though direct DNA binding of AFB1 is not possible [27]. There is evidence that AFB1-E may form such mutagenic adducts as AFB1-E-deoxyguanosine or AFB1-formamidopyrimidine (AFB1-FaPy-dG) upon rapid conjugation with N7 of guanine residues [25]. As a matter of fact, AFB1-E has been shown to naturally and unchangeably pair with residues of guanine, forming 8,9-dihydro-8-(N7-guanyl)-9-hydroxyaflatoxin B1 (AFB1-E-N7-dG) adducts that may cause DNA damage and mutations [26, 28, 29]. Many occurrence of hepatocellular cancer have been linked to the spontaneous opening of the imidazole ring or depurination of AFB1-E-N7-dG adducts yielding AFB1-FaPy-dG [29]. AFB1-E-N7-dG has higher affinity for enzymes involved in DNA repair pathways than AFB1-FaPy. It has been reported that the adduct of AFB1-FaPy-dG blocks the replication more effectively than that of AFB1-E-N7-dG, which induces at least six times more G-to-T conversion than AFB1-E-N7-dG [25]. In vivo, it would appear that AFB1-FaPy-dG is the major adduct and the cause of the most lethal impairment of replication caused by aflatoxin.

It is known that cytochrome P450 bioactivates AFB1 in human liver microsomes. When AFB1 intake is low, CYP1A2 is primarily responsible for forming AFB1-E-dG and DNA damage, whereas at higher levels, CYP3A4 is mainly responsible [30, 31]. There is evidence to suggest that 95% of AFB1-DNA adducts are formed through the action of CYP1A2 [32, 33]. According to a microarray study conducted on mice that were exposed to AFB1, almost 200 genes showed adverse effects due to the exposure, and histone transcript levels displayed a fivefold decrease as a result of the exposure. AFB1 intake is also associated with the production of serum albumin adducts and the excretion of AFB1-E-N7-guanine in the urine [34]. There are strong correlations between AFB1-E-N7-dG adducts and liver tumor incidences [35]. Therefore, the AFB1-E-N7-dG adduct is quickly washed out of DNA and eliminated by the urine system [35]. The risk of developing HCC is 9.1 times higher in people who excrete AFB1-E-N7-dG adducts than in those who do not excrete them [36]. According to these findings, the formation of HCC is profoundly affected by AFB1-DNA adducts [32].

Excess reactive oxygen species (ROS) oxidize DNA bases, contributing to DNA damage induced by AFB1 [37]. As a result of AFB1, ROS production, superoxide dismutases (SOD), glutathione peroxidases (GPx), catalases (CAT), and glutathione reductases (GR) are increased. In contrast, glutathione (GSH) is decreased, while DNA fragments, caspase-3, superoxide anion radicals, and lipid hydroperoxides are increased. AFB1-induced hepatocarcinogenesis is linked to indicators of oxidative stress and DNA damage [38, 39]. Moreover, lipid peroxidation has been shown to increase the susceptibility to mutations and trigger aldehyde-DNA adducts on the code 249 mutant site of p53 [40]. By modulating the transcription of antioxidant genes, p53 prevents further ROS/RNS formation; however, apoptosis is induced by DNA damage because it turn on the prooxidant genes. Lymphocytes in the spleen are killed when exposed to AFB1 is well correlated with elevated oxidative stress [41]. Accordingly, the TP53 mutation can be used to determine whether an individual has been exposed to AFB1 [42]. Important antioxidant enzymes such as selenium sulfide reductase, remove free radicals in response to AFB1-induced mitochondrial damage, thus reducing By activating the antioxidant system, vital oxidative damage [43]. There is an association between dietary intake of AFB1 and hepatocyte apoptosis rate [44]. As a result, AFB1 generates excess reactive species, leading to apoptosis of hepatocytes through the inability of the antioxidant system to function properly [45]. Eight-hydroxy-2′-deoxyguanosine (8-OHdG), which was present in large quantities in tissues exposed to AFB1, has been reported as being increased by AFB1 treatment [37]. When hydroxyl radicals interact with DNA's guanine residues, they produce the 8-OHdG adduct, a biomarker for oxidative DNA damage [46]. The AFB1-induced genotoxicity requires a DNA damage checkpoint response to ensure genome integrity and organism survival [47]. As a result of DNA damage, AFB1 activates multiple critical proteinsalong with enzymes for DNA repair, and p53 [48]. The activation of ATM occurs as a result of DNA double-strand breaks initiated by AFB1, which are among the earliest kinases to become activated. Interestingly, DNA double-strand breaks induced by AFB1 do not activate the ATR/Chk1 pathway [32].

DNA repair is modulated by cell cycle-dependent regulation, including activation of checkpoints, which reduces the progression of the cell cycle to initiate DNA repairing mechanisms [49]. Thus, provisional DNA injuries are kept from becoming inheritable mutations [50]. Inhibition of cell cycle checkpoints by AFB1 leads to mutations of ATM kinase [51]. DNA double-strand breaks are repaired less efficiently by AFB1 and apoptosis is decreased, resulting in higher cancer risks [52]. DNA with altered bases triggers the ATM kinase to activate signalling pathways for its repair, since ATM is a crucial component of cell cycle checkpoint activation [53]. ATM kinase bind to and regulate the SMC1 (structural maintenance of chromosome 1) protein. DNA replication forks and DNA damage response necessitate the presence of SMC1 [54, 55]. By activating p53 and inhibiting cyclin-dependent kinases, ATM signaling inhibits the progression of the cell cycle from G1 to S [56]. As a result, ATM regulates the G2/M checkpoint, which plays a crucial role in maintaining genomic integrity [57, 58]. Chk2 is also activated by ATM through phosphorylation in response to DNA damage [58]. By activating the p53 signal pathway, phosphorylated Chk2 (pChk2) induces the G2/M cell cycle checkpoint [59, 60]. As it turns out, AFB1 activates the ATM-Chk2-p53 pathway, which leads to arrest in the G2/M phase via the CDC25-cyclin B/cdc2 pathway [61]. By ubiquitinating p53 and facilitating proteasome-mediated degradation, Mdm2 contributes to this signaling pathway [62, 63]. The ATM-Chk2-p53 axis thus plays an important role in responding to DNA damage caused by AFB1.

## Coprine:

Coprine is a mycotoxin that occurs in mushrooms in the genera Coprinopsis. In this case, coprine exists in the ink-cap mushroom, Coprinopsis atramentarius, and causes a hypersensitivity to alcohol and ethanol. The coprine compound found in the mushroom inhibits the body's ability to metabolize alcohol. Alcohol metabolism in the body requires the conversion of ethanol into acetaldehyde by the enzyme alcohol dehydrogenase. Enzyme acetaldehyde dehydrogenase further breaks down the acetaldehyde into acetate [64]. In the final step of the citric acid cycle, acetic acid is converted into carbon dioxide and water.. Therefore, the coprine compound once ingested with alcohol leads to a buildup of acetaldehyde. In this case, the non-protein amino acid is converted into its metabolite, 1-aminocyclopropanol, which inhibits the functioning of the enzyme acetaldehyde dehydrogenase. Other species that contain coprine include; *Coprinopsis acuminata*, *Coprinopsis alopecia*, *Coprinopsis erythrocephala*, *Coprinopsis fusispora*, *Coprinopsis geesterani*, *Coprinopsis insignis*, *Coprinopsis jamaicensis*, *Coprinopsis krieglsteineri*, *Coprinopsis maculatus*, *Coprinopsis ochraceolanata*, *Coprinopsis romagnesiana*, and *Coprinopsis variegate*. Coprine poisoning causes facial reddening or flushing on the victim. In addition, the consumption leads to nausea and vomiting with the victim exhibiting agitations and tingling in limbs. In other instance, the victim may report severe headaches. Several studies have suggested that coprine is a potential chemopreventive agent against alcohol abuse. However, its carcinogenic, mutagenic, and male gonadotoxic effects make it unsuitable for this application [65].

## Fumonisins:

Fungi belonging to the genus Fusarium, such as Fusarium moniliforme, Fusarium proliferatum, and Fusarium verticillioides, are the sources of the mycotoxins known as fumonisins. The fungus is prevalent in corn and agricultural commodities such as rice and sorghum in the field or storage. The major types of fumonisins that pose health risk to humans and animals include fumonisin B1 (FB_1_), fumonisin B2 (FB_2_), and fumonisin B3 (FB_3_). Environmental factors such as temperature, humidity, and rainfall determine levels of fumonisin B1 (FB_1_) in corn. For instance, drought conditions followed by warm and wet weather during the flowering phase contribute to fumonisins contamination [66]. In addition, insect damage to maturing corn ears exposes the plants to environmentally present fumonisins. FB1 has been demonstrated to disrupt sphingolipid metabolism by blocking the enzyme ceramide synthase, which causes the build-up of sphinganine in cells and tissues due to their structural similarity. Fumonisins consumption in human contributes to esophageal and liver cancer while in animals fumonisins consumption induces equine leukoencephalomalacia and porcine pulmonary edema. There has been liver disease and tumors observed in rodents.

Among the mycotoxins released by *Fusarium* are fumonisins, trichothecenes, and zearalenone. The *Fusarium verticilloides* and *F. proliferatum* produce fumonisins. In 1988, they were isolated for the first time from *F. verticilloides* in South Africa. In the majority of cases, they can be found in maize, which is the main source of food for them. Due to the fact that these compounds are highly polar in nature, water is a very suitable solvent for them. The International Agency for Research on Cancer (IARC) has identified fumonisins as a group of chemicals that may cause cancer in humans.. South Africa, China and Italy have all reported cases of oesophageal cancer caused by this chemical in humans. There is general consensus that the pancreas, liver and kidneys are the organs most affected, with increased weights and necrosis as a result. A group of carcinogens known as fuminosins B1, B2, and B3 is composed of nongenotoxic carcinogens. A correlation has been found between the consumption of grains contaminated with fumonisins and an increased risk of oesophageal cancer in humans. It has been proven that fumonisin B1 causes liver and kidney dysfunction as well as oesophageal cancer, as well as spinal cord and brain damage. In terms of effects, funonicin B2 is similar to funonicin B1, but their effects are relatively mild compared to each other [67, 68].

Fumonisins are a class of compounds that have been linked to human cancer by the International Agency for Research on Cancer (IARC).. By accumulating sphinganine, free sphingoid bases cause hepatic and nephrotic toxicity, resulting in abnormal cell proliferation and apoptosis. It was found that the fumonisin B (FB) mycotoxins produced by *F. moniliforme* have the potential to induce cancer in rats because they are capable of producing rare hepatocytes which acquire resistance to the mitoinhibitory action of 2-acetyl-aminofluorene (2-AAF). In patients, sphingolipids cause neurotoxicity and pulmonary edema since they are important for normal membrane function. Serum free sphingoid base accumulation may serve as a reliable biomarker of fumonisin exposure.. The gastrointestinal tract has difficulty absorbing them, and the blood rapidly removes them. The same cannot be said for eggs, as they are neither stored well in tissues nor reported from them. A very small amount of carryover has also been observed in milk [68].

## Deoxynivalenol (DON):

Deoxynivalenol is a mycotoxin produced by the fungi *Fusarium graminearum*. Other DON producing fungi include *Fusarium culmorum*. Deoxynivalenol is widely distributed in corn and other cereal grains. Here, the organism overwinters on infected residue from the previous season's crop, which serves as an inoculum source for the current harvest.. The conditions for growth of deoxynivalenol are cool, moist conditions [69]. The fungus contaminates the crops when spores of the organism are windblown to the silks of the corn or to the anthers in the small grains. The infection is caused when the fungus gains access to the host's ear. *Fusarium graminearum* causes deoxynivalenol, a purple to pink mold that can be seen growing on the ear of corn.. Pigs may vomit once they consume the contaminated grain. In addition, deoxynivalenol acts as an immunosuppressant and in severe cases may lead to kidney problems.

Activation of the caspase-3 pathway is thought to be the mechanism responsible for DON-induced apoptosis in porcine hepatocytes [70]. In Jurkat cells, cleavage of poly (ADP-ribose) polymerase (PARP) and the activation of caspase-3 have also been observed during DON induced apoptosis [71, 72]. Nagase *et al*., (2001) have demonstrated that the caspase-9, caspase-3, and caspase-activated DNase (CAD) in HL-60 cells, when they are subjected to T-2 toxin-induced apoptosis, are activated through an increase in cytosolic release of cytochrome-c. The protein expression of caspase-3 increased with increasing DON concentration, suggesting that the toxin activated caspase-3 in HL-60 cells, which showed a substantial increase in expression following DON treatment compared to the control.

A number of studies have shown that ERK, p38, and JNK signaling pathways are involved in increased transcriptional activity [27, 73] as well as p38 mitogen-activated protein kinases [74]. Caspase-activating cascades are currently well-characterized as regulating apoptosis in two different ways: through cell death receptors and through mitochondrial dysfunction [75]. A recent study has also shown that the activation of caspases in response to T-2 toxin is associated with the release of cytochrome c from mitochondria into the cytosol, which contributes to cell apoptosis [72]. In spite of this, there is still no information about the mechanisms through which DON induces apoptosis in cells.

## Ochratoxins (OTA: The hormone disrupting mycotoxin):

*Aspergillus ochraceus* and Penicillium verrucosum are the fungus responsible for making ochratoxins. Depending on commodities and location, ochratoxin can also be produced by fungus like Aspergillus niger and Aspergillus carbonarius. Mold, dampness, and warmth are all necessary for ochratoxins to be created in large quantities during storage [76]. Visible mold from different species varies in appearance; blue green with *penicillium*, yellow tan with *Aspergillus ochraceus*, and black with *Aspergillus niger* and Aspergillus carbonarius. Ochratoxins may be present in grains that have a 'musty' odor and grains whose seed coat is compromised such as stress cracks and broken kernels. Ochratoxin consumption affects the kidney and liver. Studies have also proved that the mycotoxin is carcinogenic to rats and mice. It has been very rare to find ochratoxin in Asia due to the presence of *Aspergillus ochraceus* and *Penicillium verrucosum*. The presence of this compound is generally found in cereals, beans, and other plant products. Coffee is one of the most little-known sources of food borne mycotoxins. As a nephrotoxic mycotoxin, OTA is one of the most dangerous crop storage mycotoxins. Ochratoxin (OTA) comes in four different varieties: A, B, C, and D. One of the most prevalent and toxic ochratoxin compounds is ochratoxin A (OTA) [77].

In addition to affecting kidney function, it affects the nervous system as well. Human beings can develop breast cancer as a result of DNA damage due to the OTA. Humans have had fibroadenomas described as a form of breast cancer for quite some time; however, it is possible that the development of the same type of cancer in mice and rats may be linked to the appropriate administration of OTA. According to the International Agency for Research on Cancer (IARC), OTA appear to be possible carcinogens that can contribute to the development of cancer in humans.

It is known that ochratoxin is taken in by the digestive system, typically by thethe jejunum. A rapid binding occurs between ochratoxin and serum albumin as soon as it reaches the bloodstream. In the body, it circulates as a serum-protein adduct as it travels through different parts of the body. Biomarkers for ochratoxin exposure could be derived from this adduct. It has been found that ochratoxin is a nephrotoxic mycotoxin that causes deadly kidney illnesses that cause the organs to shrink and develop tubules. Several studies have shown that it is responsible for the development of porcine nephropathy, a condition which is widely studied. There is a high concentration of ochratoxin in the kidneys, which is followed by the liver, the muscles and the fat in the body and is being transferred in humans through the meat. In addition to this, ochratoxin toxicity also causes an increase in water consumption, a malfunctioning bladder, and diarrhea as a result of blood leaking into urine and faeces [77].

In humans and animals, ochratoxins disrupt hormone levels. A study was conducted in 2011 in New Jersey to screen urine samples from 9- to 10-year-old Jersey girls. Those girls who tested positive tended to be shorter and were less likely to have reached the puberty onset than those who tested negative. In addition, it has also been found that female piglets are capable of displaying estrogenic properties when they are fed with OTA grains that are contaminated [78]. The isocoumarin moiety in ochratoxin is linked with a peptide bond to phenylalanine. As a metabolic inhibitor, ochratoxin inhibits the synthesis of phenylalanine as well as the conversion of other substances to phenylalanine. The process is inhibited because enzymes required for synthesis are not synthesized. Inhibiting the production of phenylalanine into tyrosine and the formation of the phenylalanine-tRNA complex are examples of how it inhibits the action of phenylalanine. Additionally, it hinders the production of ATP. This promotes membrane lipid peroxidation, the source of superoxide and hydrogen peroxide radicals..

## Trichothecenes:

This is a mycotoxin from the fungal metabolites identified as trichothecenes. In this case, the trichothecenes fall into two categories that is macrocyclic and non-macrocyclic trichothecenes. Within the non-macrocyclic class, the trichothecenes are classified as either Type-A or Type-B. Mycotoxins like T-2 toxin and HT-2 toxin are found in Type-A trichothecenes, along with diacetoxyscirpenol (DAS). The *Fusarium sporotrichioides* fungus is responsible for making T-2 toxin.. The toxin is present in maize, barley, oat, and rice. T-2 toxin is naturally present in Asia, Africa, South America, Europe, and North America [79]. The toxin occurrence increase in conditions with increased humidity and temperatures ranging between 6 to 24°C. The mycotoxin is prevalent in grain related poultry and animal feeds. Storage conditions have to be below 14% moisture and keep the grains free from insect damage. Furthermore, the grains have to be properly dried prior to storage to minimize the chances of contamination with T-2 toxin. Protein synthesis is inhibited by T-2 toxin and other trichothecenes, which has a knock-on effect on DNA and RNA synthesis. Mycotoxins also have an effect on rapidly dividing cells, including those lining the digestive tract, the skin, and the erythroid organs.. Animals display weight loss or poor weight gain, bloody diarrhea, and hemorrhage. In other instances, there may be reduced immunity due to a decrease in antibody levels and immunoglobulins. Poultry and cattle may have decreased production of eggs and milk due to ingestion of the T-2 toxin.

 A total of 175 trichothecenes have been isolated so far, but only a few of them have been found to contaminate foods such as wheat and rice. Types A and B of *Fusarium* spores can be classified into two main groups, these examples being derived from different species of *Fusarium*. T-2 toxin, HT-2 toxin, and Di Acetoxy Scirpenol (DAS) are all examples of type A trichothiecenes, whereas De-Oxy Nivalenol, Nivalenol (NIV), and fusarenol X are all examples of type B trichothiecenes.. For biological warfare, these are the most preferred weapons. Trichothecenes caused alimentary toxic aleukia (ATA) in 1950, causing nearly 1 lakh deaths. T-2 toxin is found responsible for ATA. The syntheis of RNA and DNA are found inhibiting by T-2 toxin as well as it also reported to inhibit cell apoptosis [80, 81, 82]. As a result of animal experiments, it has been proved that inhalation is 40 times more dangerous than ingestion when it comes to these trichothecnes.. Fortunately, they are not found in milk, but they are accumulated in tissues or eggs. In most cases, the symptoms of toxicity are nausea, vomiting, and anorexia. In eukaryotic cells, these compounds can cause cell lysis, inhibition of mitosis, and an increase in the number of divisions per cell. The drug also interferes with the normal apoptosis of the cells and the immune system by reducing the number of leukocytes. According to research conducted by Marasas and colleagues, trichothecenes have been linked to an increase in cases of esophageal cancer in the Republic of Transkei in South Africa. However, it should be stressed that ATA has never been documented in Asia [82].

The fungus *Fusarium* which consists of species F-sporotrichioides, F-langsethiae, F-acuminatum, and F-poae, produces T-2 toxin, which is one of the trichothecenes. It is known that these fungi are found in moldy whole grains of wheat, barley, and oats of poor quality. The half-life of T-2 toxin is very short. This toxin is commonly known as "yellow rain" and used as a chemical weapon. People who have been infected with the T-2 toxin are frequently found to have antibodies against the toxin in their blood. It is capable of inhibiting DNA and RNA synthesis and is capable of causing cells to undergo apoptosis in response to it. It is worth noting, however, that *in-vivo*, the toxin undergoes a rapid metabolization and becomes HT-2 mycotoxin. A common symptom of ATA is high fever and bleeding of the skin, nose, throat, and gums, as well as necrosis and a failure of immunity. Radiation poisoning symptoms may sometimes mimic these symptoms. A T-2 mycotoxin-dose-dependent breast cancer model was also developed [83].

The fungus *Fusarium cerealis* releases another trichothecene called nivalenon. The toxin is found in wheat, maize, oat, barley, and rye. Bone marrow toxicity is caused by it. A study in HL-60 cells found that recombinant DNA induced chromosomal aberrations and sister chromatid exchange with DNA damage and DNA strand breaks was slightly increased *in-vitro*, as well as apoptosis was innhibited. In cultured human lymphocytes, it inhibits the process of blastogenesis as well and faeces are excreted as a result of this process. In the same way, verrucarin, a trichothecene produced by *Fusarium* species and *Myrothecium verrucaria*. In mice, it was found to lead the development of breast cancer. In breast cancer cell lines such as, MDA-MB-231 and T47D, the protein synthesis inhibitor verrucarin A was shown to induce growth inhibition and apoptosis. In MCF-7, it alters the cell cycle regulatory proteins and triggers apoptosis by activating P-38 MAPK which is dependent on reactive oxygen species [84, 85, 86, 87, 88].

##  Zearalenone:

Fusarium graminearum is a fungus that produces the mycotoxin zearalenone.. This mycotoxin appears as a greenish fluorescent compound when seen under short-wavelength ultraviolet light on thin-layer chromatographic plates. It is often found in corn and other crops such as wheat, sorghum, and barley [89]. The Fusarium genus thrives in damp, chilly environments, where it may easily spread and infest crops. Mammal precocity is caused by the mycotoxin zearalenone. Also, it is been shown to cause hormonal alterations like hyperestrogenism in pigs because of the way it interacts to estrogen receptors. The warm and temperate regions are home of zearalenone. *Fusarium graminearum*, *Fusarium cerealis*,*Fusarium equiseti*, *Fusarium incarnatum* and *Fusarium culmorum* release this toxin on cereal crops. *Fusarium* fungus thrives in fields treated with an herbicide called "Round Up" containing glyphoset. As a result, it has been classified as a potential carcinogen since it damages DNA. Protein biosynthesis is abnormally activated by it. The mycoestrogens produced by zearalenone are hyperestrogenic. Estradiol and zearalenone are similar in their estrogenic properties. The female sex hormone estradiol belongs to the estrogen family [90, 91]. Among nine to ten year old girls in New Jersey, USA, urinary mycoestrogen levels were examined. Mycoestrogens were found in the urine of 86% of the female participants who ate popcorn or beef. Girls with ZEA were said to be shorter and have less developed breasts. U.S. animals have been fed "Zeranol," a ZEA derivative, since the 1970s. It was swiftly outlawed in the EU and many other regions [78].

## Gyromitrin:

Gyromitrin is a mycotoxin produced by fungus of the genus *Gyromitra* such as *Gyromitra esculenta*. Other species harboring the toxin include *Gyromitra gigas* and *Gyromitra* fastigiata. In this case, the Gyromitrin containing mushroom species are known as the false morel. After consumption, gyromitrin compound is converted into monomethyl hydrazine (MMH) in the acid environment of the stomach and passage through the liver [92]. Consumption of gyromitrin toxin causes significant hepatotoxicity that may lead to neurotic seizures in severe cases. Other symptoms may include delayed nausea, vomiting, and diarrhea. In more severe cases, the liver may be damaged leading to jaundice which may lead to liver failure within 48 to 72 hours.

In addition to acute poisoning, false morel consumption is associated with a high risk of cancer. The edible wild mushroom *G. esculenta* contains gyromitrin, acetaldehyde N-methyl-N-formylhydrazone, and N-methylhydrazine. In mice and hamsters, N-methylhydrazine is an antagonistic compound that induces tumors through an intermediate compound, N-methyl-N-formylhydrazine. Moreover, upon administration of gyromitrin to a mouse stomach, methylhydrazine is observed to be formed in the stomach of the mouse. According to these findings, consumption of *G. esculenta* may present a potential hazard to health both in terms of a carcinogenic effect as well as acute toxicity.

## Rubratoxin:

*Penicillium rubrum* and *Penicillium purpurogenum* are two species of fungi that produce rubratoxin, a substance commonly found in cereal grains. The toxin is found to causes liver cancer in some people. A rubratoxin can be classified as either A or B based on the type of toxicity it causes. There is no doubt that Rubratoxin B if consumed in high doses can cause liver damage and teratogenicity, as it induces nuclear and internucleosomal fragmentation in the body. Apoptosis is a consequence of the loss of the P-53 gene in P-53 null cells [93].

## Citrinin (CIT):

Plant products such as grains, fruits, and vegetables often contain citrinin. It is nephrotoxic, hepatotoxic, cytotoxic, and genotoxic. Citrinin is responsible for oxidative DNA damage. Apple juice and grape juice contain citrinin produced by *Penicillium expansum*. Pepeljnjak *et al*. (2002) showed that *Penicillium* species isolated from decaying apples are citrininotoxinogenic. Small adenomas form in the livers and kidneys of rats fed citrinin on a regular basis. The fact that it causes chromosomal aberrations and hypodiploidy in the bone marrow of mice is further evidence that it is carcinogenic.. Citrinin exposure can be measured in urine, and kidney is the main target of citrinin exposure [ 95, 96, 97, 98, 99].

## Patulin (PAT):

Many molds produce patulin, including *Aspergillus*, *Penicillium expansum*, and *P. griseofulvum*. It is most common in rotting apples and goods made from them.. This is commonly found, as a mycotoxin, patulin (4-hydroxy-4H-furo [3,2c] pyran, 2[6+1]-one) which is mutagenic and genotoxic, causing oxidative DNA damage and cancer in humans and animals. Upon reflection and transparency of light, patulin appears as a yellow spot under visible light, whereas under long wavelength ultraviolet light, patulin appears as a yellow-orange, fluorescent spot. It has long been documented that patulin causes fibroadenomas in the breasts of humans. Additionally, it causes colorectal cancer. Contact with rotting fruits is the primary source of the PAT that causes skin cancer. In PAT-induced skin cancer, inhibitory phosphorylation of BAD may interfere with apoptosis.. Apples and pears, as well as products made from them, contain the most patulin. In humans and animals, patulin also causes neurotoxicity, immunotoxicity and gastrointestinal effects. In recent years, most juice producers have been using rotten apples and pears, containing patulin, which causes birth defects and cancer. Unknowingly, juice lovers consume strong disinfectants as they wash rotten fruits immediately during the process in order to stop the deterioration process [95, 100, 101, 102, 103, 104, 105, 106].

## Alternariol (AOH) and Alternariol Monomethyl Ether (AME):

In *Alternaria alternata* and *A. solani*, AOH and AME mycotoxins are found.. Mucoestrogens and cholinesterase enzyme inhibitors have been reported to function in alternariol toxin. Furthermore, AME is also widely distributed throughout the world and shows it carcinogenic and mutagenic, leading to very high incidence rates of oesophageal cancer. DNA breaks are also caused by it. Furthermore, AME was found to be a strong inhibitor of topoisomerase I and II, which contributed to its genotoxicity [107, 108, 109].

## Oxalic acid:

*Aspergillus* and *penicillium* produce oxalic acid, which is a mycotoxin. Human pulmonary aspergillosis is caused by Aspergillus niger in the lungs, causing high levels of oxalic acids to be formed by the fungus, that is highly lethal to the blood vessels. As a result of the constriction of the vessels, the fatal hemorrhage may occur. During the calcification of breast tissues, crystals of oxyalic acid create calcium oxalates, molecules that bind to calcium. It is also theorized that huge quantities of these crystals collected in the breast and lungs can lead to cancer in people. Patients with A. niger infections have oxalic acid in their sputum or lung specimens. Breast cancer can be attributed to oxalic acid since humans cannot produce it themselves [110, 111, 112].

## Conclusion:

We document known data on the mycotoxin source and its exposure causing human hazards leading to mycotoxicoses.

## References

[1] Bakker MG (2018). Can J Plant Pathol.

[2] Olalekan Adeyeye  SA (2020). Crit Rev Food Sci Nutr.

[3] Alshannaq A (2017). Int J Environ Res Public Health.

[4] Bertuzzi T (2019). Toxins (Basel).

[5] Kagot V (2019). Toxins (Basel).

[6] Munkvold GP (2019). Corn Chemistry and Technology.

[7] Balendres MAO (2019). Foods.

[8] Venkatesh N, NP Keller (2019). Front Microbiol.

[9] Sun XD (2017). J Food Sci.

[10] Adeyeye SA (2016). Cogent food agric.

[11] Moretti A (2017). Methods Mol Biol.

[12] Medina A (2017). Fungal Biol Rev.

[14] Jaynes WR (2007). Appl Clay Sci.

[15] Klich MA, J Pitt (1988). Transactions of the British Mycological Society.

[16] Iqbal SZ (2014). Food Chem.

[17] Zorzete P (2013). Food Res Int.

[18] Pitt J (2000). Med Mycol..

[19] Yiannikouris A, J Jouany (2002). INRAE Productions Animales.

[20] Alvarez CS (2020). Sci Rep.

[21] Benkerroum N (2020). Int J Environ Res Public Health.

[22] Engelhart S (2002). Appl Environ Microbiol.

[23] Bertuzzi T (2017). Toxins (Basel).

[24] Zhang D (2013). PLoS One.

[25] Purchase IF (1973). Toxicol Appl Pharmacol.

[26] Fujii K (1976). Cancer Res.

[27] Moon Y (2002). Toxicol Sci.

[28] Cameselle C (1998). Bioprocess Eng.

[29] Currie JN (1915). The Journal of Biological Chemistry.

[30] Wang JS (1999). Mutat Res.

[31] Yassin MA (2015). Italian Journal of Food Science.

[32] Pitt  JI (1987). Appl Environ Microbiol.

[33] B&era EV (2011). Sci Total Environ.

[34] Gelderblom WC (1988). Appl Environ Microbiol.

[35] Goel S (1996). Vet Hum Toxicol.

[36] Stockmann-Juvala H (2008). Hum Exp Toxicol.

[37] Marasas WF (1984). Int J Cancer.

[38] Merrill AH  Jr (2001). Environ Health Perspect.

[39] Riley RT (2001). Environ Health Perspect.

[40] https//www.jstor.org/stable/42932589.

[41] Li Y (2011). J Agric Food Chem.

[42] Yuan G (2014). J Environ Sci (China).

[43] Marasas WF (1979). J Agric Food Chem.

[44] Dashek WV (1986). Bioessays.

[45] Mughal MJ (2017). Oncotarget.

[46] Pestka JJ (2008). Toxicol Sci.

[47] Tsuda S (1998). Mutat Res.

[48] huv&er AC (1999). Food Chem Toxicol.

[49] Brera C (2006). J Agric Food Chem.

[50] https//www.grafiati.com/en/.

[51] Scott PM (2012). Mycotoxin Res.

[52] Man Y (2017). Toxins (Basel).

[53] Kurz EU, SP Lees-Miller (2004). DNA Repair (Amst).

[55] Lee JH, TT Paull (2007). Oncogene.

[54] https://pubmed.ncbi.nlm.nih.gov/16869743/.

[56] Niida H (2006). Mutagenesis.

[57] Kudoh A (2005). J Biol Chem.

[58] Wilson KA (2008). Cell Cycle.

[59] Hirao A (2000). Science.

[60] Yu Q (2002). Cancer Res.

[61] Yin H (2016). Oncotarget.

[62] Chen X (2013). PLoS One.

[63] Noon AP (2010). Cancer.

[64] Barac A (2019). Clinically Relevant Mycoses.

[65] https//www.ncbi.nlm.nih.gov/books/NBK493175/.

[66] Luo YX (2018). Food control.

[67] Lin YC (2014). J Biol Chem.

[68] Ajiboye TO (2016). Pharm Biol.

[69] Tola M, B Kebede (2016). Cogent Food & Agriculture.

[70] Mikami O (2004). Toxicology.

[71] Shifrin VI (1999). J Biol Chem.

[72] Nagase M (2001). Biosci Biotechnol Biochem.

[73] Bondy GSJ (2000). Toxicol Environ Health B Crit Rev.

[74] Yang GH (2000). Toxicol Appl Pharmacol.

[75] Budihardjo I (1999). Annu Rev Cell Dev Biol.

[76] Perrone G (2020). Microorganisms.

[77] Gursoy-Yuzugullu O (2011). Liver Int.

[78] Groopman JD (1992). Carcinogenesis.

[79] Pfliegler WP (2019). Front Microbiol.

[80] Weng MW (2017). Oncotarget.

[81] Chen J (2016). Toxicol Ind Health.

[82] Hussain SP (2007). Oncogene.

[83] Yang J (2012). Poult Sci.

[84] Liu W (2018). Environ Pollut.

[85] Fasullo M (2008). Mol Carcinog.

[86] Yang X (2013). Toxicol Appl Pharmacol.

[87] Burma S (2001). J Biol Chem.

[88] Sancar A (2004). Annu Rev Biochem.

[89] Ji C Y  Fan, L Zhao (2016). Anim Nutr.

[90] Warmerdam DO (2010). Mutat Res.

[91] Yang XH (2006). Results Probl Cell Differ.

[92] Huang D (2019). Food Chem Toxicol.

[93] Nagashima H (1998). JSM Mycotoxins.

[95] Brause AR (1996). J AOAC Int.

[96] Flajs D, M peraica (2009). Arh Hig Rada Toksikol.

[97] Arai M, T Hibino (1983). Cancer Lett.

[98] Jeswal P (1996). Cytobios.

[99] Ali N M (2015). Arch Toxicol.

[100] Kwon O (2012). Cell Signal.

[101] https//www.worldcat.org/title/perishables-handling-quarterly/oclc/223560309.

[102] Welke JE (2011). Braz J Microbiol.

[103] Liu BH (2003). Toxicol Appl Pharmacol.

[104] Glaser N , H Stopper (2012). Food Chem Toxicol.

[105] Saxena N (2011). Toxicol Appl Pharmacol.

[106] Guo X (2013). Cell Death Dis.

[107] Branzei D, M Foiani (2008). Nat Rev Mol Cell Biol.

[108] Bartek J, J Lukas (2007). Current opinion in cell biology.

[109] Lavin MF, S Kozlov (2007). Cell cycle.

[110] Shimada T, FP Guengerich (1989). Proc Natl Acad Sci U S A.

[111] Lin YC (2016). Proc Natl Acad Sci U S A.

[112] Gallagher EP (1996). Toxicol Appl Pharmacol.

